# Supplementary material for: 1/4 is the new 1/2 when topology is intertwined with Mottness
Source: Nat Commun. 2023 Sep 26;14:5999. doi: 10.1038/s41467-023-41465-6 (PMC10522641; doi:10.1038/s41467-023-41465-6)
Supplement: Supplementary file 1 — Supplementary Information [file 41467_2023_41465_MOESM1_ESM.pdf]

Supplementary information

Peizhi Mai[1], Jinchao Zhao[1], Benjamin E. Feldman[2,3,4] and Philip W. Phillips[1,†]
[1]*Department of Physics and Institute of Condensed Matter Theory, University of Illinois at Urbana-Champaign, Urbana, IL 61801, USA*
[2]*Geballe Laboratory of Advanced Materials, Stanford, CA 94305, USA*
[3]*Department of Physics, Stanford University, Stanford, CA 94305, USA and*
[4]*Stanford Institute for Materials and Energy Sciences, SLAC National Accelerator Laboratory, Menlo Park, CA 94025, USA*
(Dated: July 2022)

OVERVIEW

In this supplement, we provide further information to support our conclusion for the interaction-driven topological Mott semimetal (TMSM) and insulator (TMI) with quantum anomalous Hall (QAH) effect in a general strongly correlated quantum spin Hall (QSH) system. In the following, we first include the details of determinantal quantum Monte Carlo (DQMC) and dynamical cluster approximation (DCA) simulations. Then we have three sections of supplemental results for the Kane-Mele-Hofstadter-Hubbard (KM-HH) and Bernevig-Hughes-Zhang-Hofstadter-Hubbard (BHZ-HH) from DQMC and DCA simulations as well as Hatsugai-Kohmoto (HK) model from analytical calculations. At the end, we also have a section to discuss how the bilayer flat-band KM-HH model explains and predicts the experiments.

DETAILS OF DQMC SIMULATIONS

We use the DQMC code in https://github.com/edwnh/dqmc. The Hubbard-Stratonovich transformation is not SU(2) symmetric. We discretize the imaginary time β into L slides with $\Delta\tau = 0.1$. We conduct 5000 warmup sweeps and 200000 measurement sweeps (10 measurements per sweep) at each Markov chain. We scan the chemical potential from -10 to 10 (about 160 values) to obtain the density dependence of physical quantities. Depending on the sign problem in Supplementary Fig. 1 for example (little change under finite magnetic field), we use different numbers (from 2 to 40) of Markov chains to bring down the error bar.

DETAILS OF DCA SIMULATIONS

We use the DCA++ package[1] in https://github.com/CompFUSE/DCA. We use the continuous-time, auxiliary-field quantum Monte-Carlo algorithm[2, 3] as the cluster solver for DCA simulations. The expansion order of the CT-AUX QMC algorithm is typically 50-200. Increasing this number can alleviate the sign problem to some extent but that also means doing more measurements effectively. Depending on the sign problem for a given parameter set, we measure $(1 \sim 4) \times 10^7$ samples for the correlation functions. Six to eight iterations of the DCA loops are typically needed to obtain good convergence for the DCA self-energy and chemical potential for the measurement of the gap.

SUPPLEMENTAL DCA AND DQMC RESULTS FOR THE KM-HH MODEL

Sign problem of DQMC simulations for the KM-HH model

The DQMC simulation for KM-HH model suffers from severe sign problems in certain doping densities. For this reason, our computation is limited to high temperatures. The average signs for the flat-band case $\psi = 0.81$ (in the unit of π) at $U = 3, \beta = 7$ and the dispersive case $\psi = 0.5$ at $U = 12, \beta = 3$ as a function of density at the zero field are shown in Supplementary Fig. 1. Note that in the flat-band case (Supplementary Fig. 1a), half-filling is not sign-problem-free due to the lack of particle-hole symmetry. This symmetry is retained in the original KM case (Supplementary Fig. 1b) and thus the average sign is 1 at $\langle n \rangle = 2$.

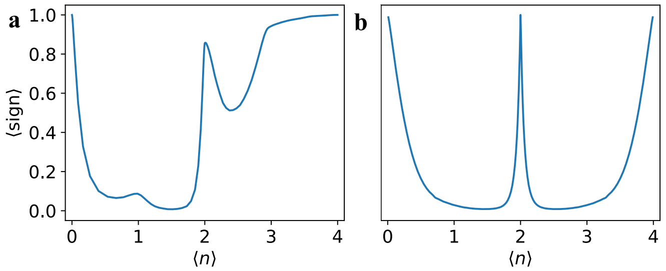

Supplementary Figure 1: The average sign for the KM-HH model with zero external magnetic flux for **a** $t' = 0.3, \psi = 0.81, U = 3, \beta = 7$ and **b** $t' = 0.3, \psi = 0.5, U = 12, \beta = 3$.

Non-interacting results for the KM-HH model at lower temperatures

Here we show the non-interacting compressibility and TRI compressibility for the KM-HH model at lower temperature $\beta = 20$ in Supplementary Fig. 2 for the flat-band case (a) and the original dispersive case (b). In either case, the integer quantum Hall effects become sharper at this lower temperature. For the TRI compressibility, the inverse slope of the leading middle line crossing $\langle n \rangle = 2$ gives the spin Chern number $C_s = 2$.

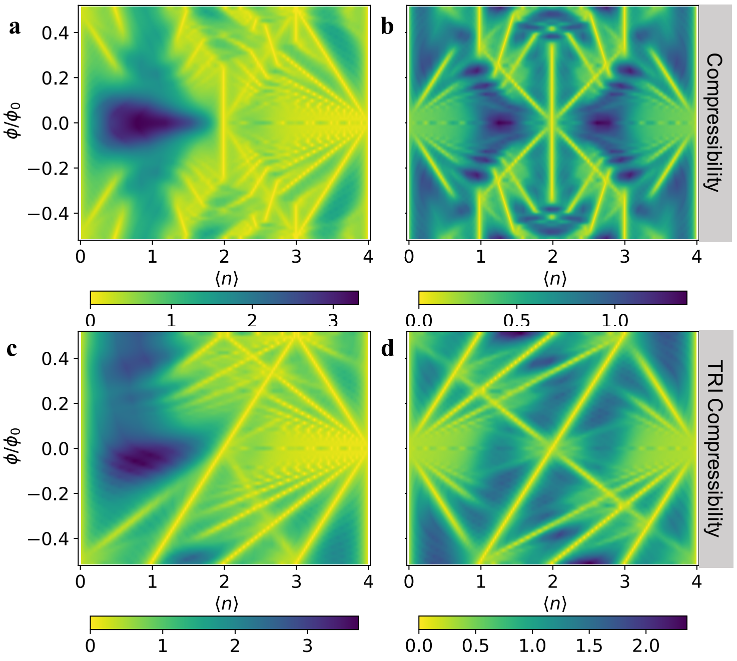

Supplementary Figure 2: The compressibility (first row) and TRI compressibility (second row) for the non-interacting KM-HH model for (**a**,**c**) $t' = 0.3, \psi = 0.81$ and (**b**,**d**) $t' = 0.3, \psi = 0.5$. The inverse temperature for both cases is $\beta = 20$.

The crossover from metal to QAH/QSH features at high temperatures

If the temperature is too high, we would not observe the non-trivial topology emerging at quarter-filling, no matter how large U is. In the Fig. 1 of the main text, at $U = 12$, we need to reach $\beta = 3$ to discern the dip in the compressibility and hence the QSH feature. If for a smaller U, we can expect the onset temperature for such a crossover (from a featureless metal to a high-temperature QAH/QSH effect) to decrease. For example, Supplementary Fig. 3 shows that $\beta = 4$ is needed to observe the high-temperature QAH behavior at $U = 3$.

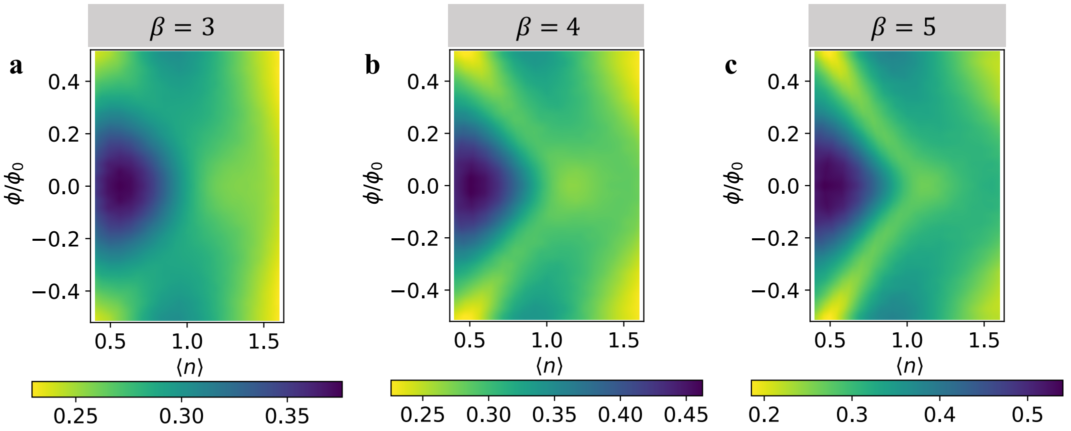

Supplementary Figure 3: The compressibility for the generalized flat-band KM-HH model ($t' = 0.3, \psi = 0.81$) as a function of magnetic flux and density with fixed for different temperatures **a** $\beta = 3$, **b** $\beta = 4$, **c** $\beta = 5$.

The crossover from QAH to QSH features at high temperatures

In Fig. 1 of the main text, we jump directly from the QAH effect at $U = 3t$ (second row) to the QSH effect at $U = 12t$ (third row) and also change the temperature. Here we fill in the gap to see how this happens by gradually increasing U while keeping $\beta = 3/t$ in Supplementary Fig. 4. As U increases, the right Landau levels start to appear while the left Landau levels become more prominent, suggesting a crossover instead of a transition.

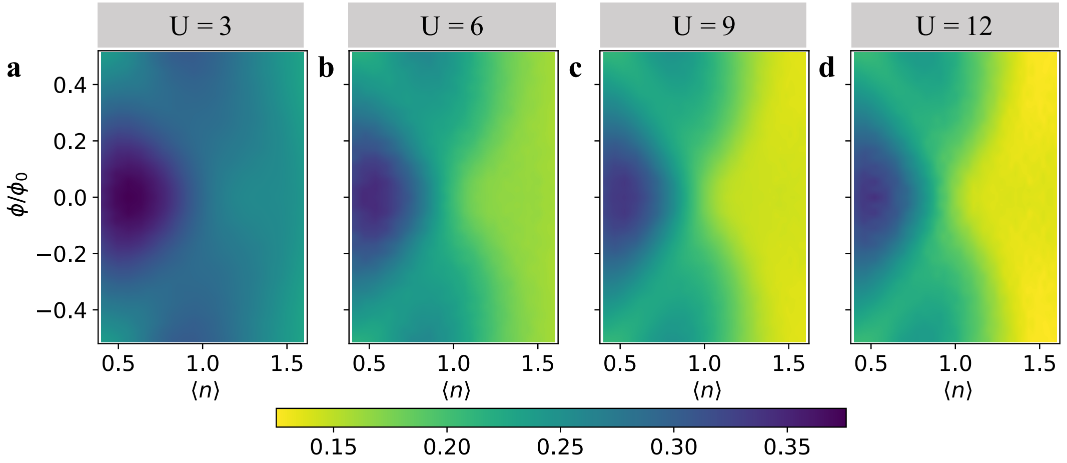

Supplementary Figure 4: The compressibility for the generalized flat-band KM-HH model ($t' = 0.3, \psi = 0.81$) as a function of magnetic flux and density at **a** $U = 3$, **b** $U = 6$, **c** $U = 9$ and **d** $U = 12$. The inverse temperature for all cases is $\beta = 3$.

The comparison between DCA and DQMC simulations

In Supplementary Fig. 5, we show a good benchmark between DCA and DQMC on the $\langle n \rangle$ versus μ relation for the KM-Hubbard model ($t' = 0.3, \psi = 0.63$) at $U = 2, \beta = 8$ and $U = 5, \beta = 4$, supporting Fig. 5 in the main text.

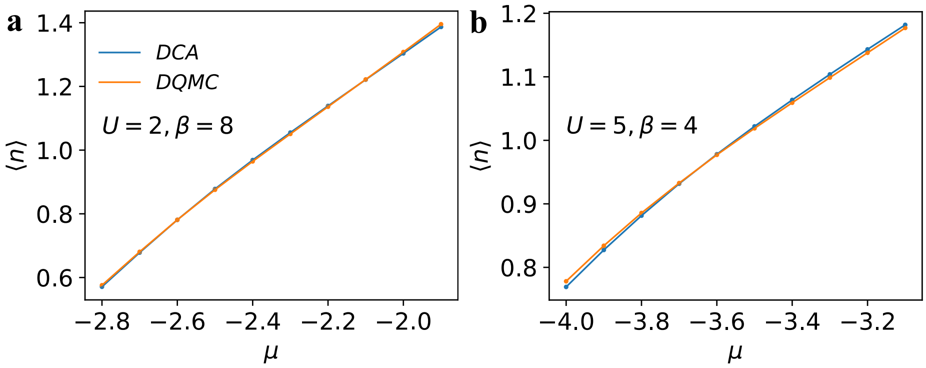

Supplementary Figure 5: The comparison on $\langle n \rangle$ versus μ relation between DQMC on a $6 \times 6 \times 2$ cluster and DCA on a $2 \times 2 \times 2$ cluster at for the generalized KM-Hubbard model ($t' = 0.3, \psi = 0.63$) at **a** $U = 2, \beta = 8$ and **b** $U = 5, \beta = 4$.

DQMC results for the original (dispersive) KM-HH model

Here we show the DQMC simulation results in Supplementary Fig. 6 for the original (dispersive) KM-HH model at $t' = 0.3, \psi = 0.5$. Comparing panels (a) and (e), we find that the strong correlation induces non-trivial topology at zero-field for $1/4-$ and $3/4-$filling with a "TRI" Chern number $C^{\mathrm{TRI}} = 1$, which is akin to that obtained for the spinful Haldane-Hofstader-Hubbard model[4]. This means QSH effects emerge at the zero-field $1/4-$ and $3/4-$filled KM-HH systems with a spin Chern number $C_s = 1$. The compressibility and spin susceptibility for the KM-HH model in Supplementary Fig. 6(f,g) are essentially the same as those for the BHZ-HH model in Fig. 3(b,c) of the main text. In the presence of strong electron-electron interaction, the compressibility displays a pair of zero-mode LLs at quarter-filling where the spin susceptibility has ridges. The relatively noticeable differences between the KM-HH and BHZ-HH models lie in the magnetization at the band edge as is evident from panels Supplementary Fig. 6h relative to Fig. 3d in the main text. Note the sign of the magnetization is not particularly important as a discrepancy already arises with the non-interacting case and hence likely arises from the difference in the lattices. In short, we observe the same QSH effects driven by Mottness in the quarter-filled KM-HH model with large enough Hubbard interaction and adds to the ubiquity of this correlation-driven effect. Unlike the flat-band case, here $W_{-0} = \Delta = 2$. There is no such intermediate region of U where a QAH effect emerges at high temperatures as in Fig. 1(d-f). As U becomes sufficiently large, the QSH effect appears.

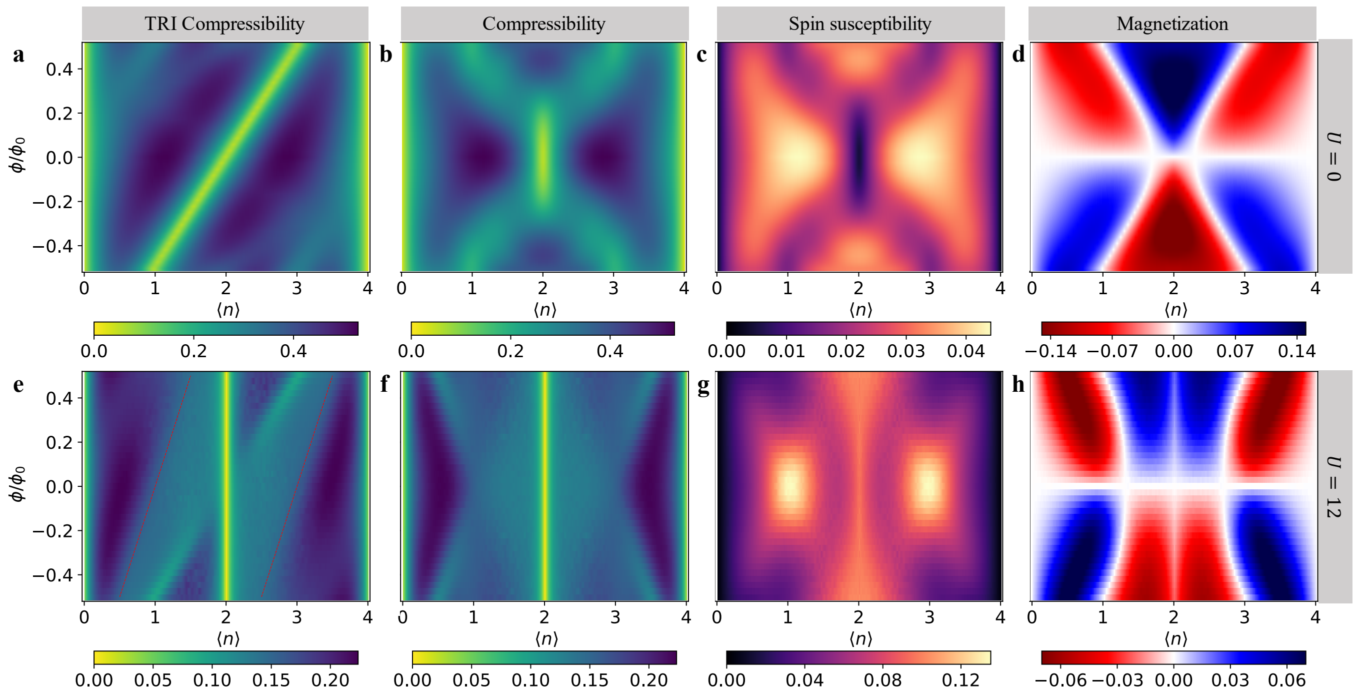

Supplementary Figure 6: DQMC results for the KM-HH-TRI and KM-HH models at $U = 0$ (first row) and $U/t = 12$ (second row). The first column shows the compressibility χ as a function of magnetic flux and electron density for the KM-HH-TRI model. The second to fourth columns show χ, spin susceptibility χ_s and magnetization $\langle m_z \rangle$ respectively, for the KM-HH model. The temperature is $\beta = 3/t$.

SUPPLEMENTAL DQMC AND DCA RESULTS FOR THE BHZ-HH MODEL

Sign problem of DQMC and DCA simulations for the BHZ-HH model

Here we calculate the average sign from DQMC simulations for the BHZ-HH model at zero field in Supplementary Fig. 7(a,b). An interesting observation is that half-filling is not sign-problem-free, unlike the KM-Hubbard case in Supplementary Fig. 1b. Previous studies[5–7] on the BHZ-Hubbard model at half-filling used dynamical mean field theory (DMFT) and found no sign problem when using quantum Monte Carlo as the impurity solver. To make a connection with these results, we further check the average sign in DCA simulations at different cluster sizes shown in Supplementary Fig. 7c. DCA is a cluster version of DMFT and DCA reduces to DMFT when the cluster size $N = 1$. In Supplementary Fig. 7c, we find that in the DMFT limit ($L = 1$), half-filling is exactly sign-problem-free, agreeing with the previous study. However, its average sign decreases dramatically as L increases (even at $L = 2$ the average sign deviates slightly from 1). Another observation is that if we turn on a staggered sublattice potential C_ν in the KM-Hubbard model, there would be a sign problem at half-filling despite time-reversal symmetry. Note that all previous quantum Monte-Carlo studies[8, 9] focused on the half-filled KM-Hubbard model with $C_\nu = 0$ and found it to be sign-problem-free.

The explanation for this is as follows. Recall that a famous sign-problem-free example is the half-filled single-band Hubbard model on a square lattice with only nearest-neighbor hopping. This arises because the Hamiltonian is unchanged under a particle-hole transformation $c_{j\sigma} \to d^\dagger_{j\sigma}(-1)^j$ with $j = \pm 1$ for sub-lattice A and B. This particle-hole symmetry is broken if we introduce the next-nearest-neighbor hopping t' which leads to a minus sign under this transformation. In the KM-Hubbard model with $C_v = 0$, the Hamiltonian under this transformation is equivalent to flipping a spin ($\uparrow\to\downarrow, \downarrow\to\uparrow$). The Hamiltonian is effectively unchanged regarding the sign problem. If C_v is finite, then it acquires a minus sign under this transformation and thus the Hamiltonian becomes different. Therefore, a sign problem is present in Supplementary Fig. 8 for the half-filling KM-Hubbard model when C_v is finite, despite the fact that time-reversal symmetry is still maintained. In the BHZ-Hubbard model, the transformation involving $j = \pm 1$ involves different orbitals rather than sub-lattices because each unit cell contains two orbitals. Then under this particle-hole transformation, the diagonal term $M + t\cos(k_x) + t\cos(k_y)$ changes a sign, leading to a change in the Hamiltonian. Due to the lack of this symmetry, there is a sign problem at half-filling as shown in Supplementary Fig. 7 for DQMC simulations. That being said, this problem may be studied using the sign-problem-free quantum Monte Carlo method in Majorana representation[10].

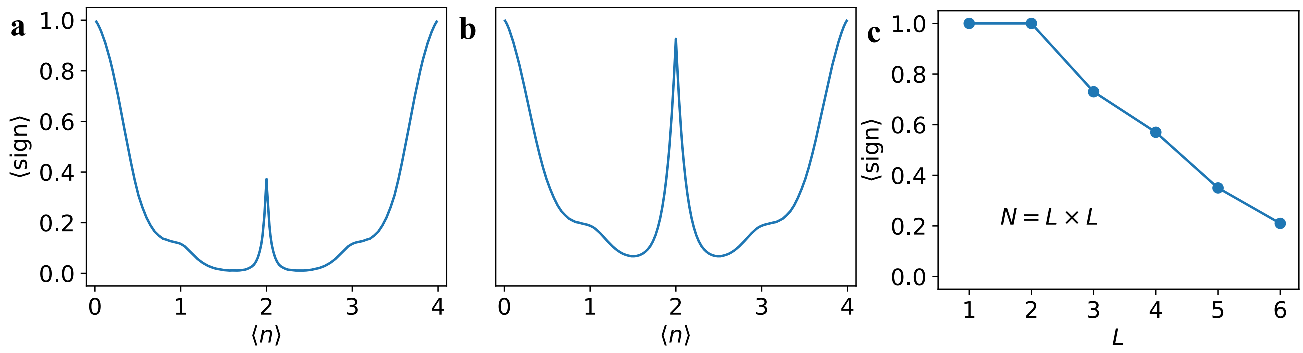

Supplementary Figure 7: Average sign of DQMC simulations as a function of density for the BHZ-HH model ($M = 1$) at zero field and **a** $U = 8$,$\beta = 4$; **b** $U = 12$,$\beta = 3$. The DQMC simulation is conducted on a $N = 6 \times 6$ cluster. Panel **c** shows the average sign of DCA simulations as a function of cluster size at half-filling and $U = 8, \beta = 5$.

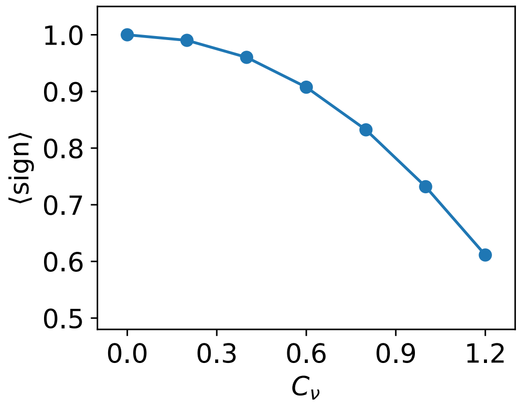

Supplementary Figure 8: Average sign of DQMC simulations as a function of a staggered sublattice potential for the KM-Hubbard model ($t' = 0.3, \psi = 0.5$) at $U = 8$ and $\beta = 4$.

The analysis of finite size effect and gap opening for the BHZ-HH model with comparison to exact diagonalization

We conduct DQMC on a 6×6 cluster. This small cluster size would suffer finite-size effect at low temperatures. An effective way to overcome it is through a minimal finite magnetic flux. An example of a finite cluster calculation for the non-interacting BHZ-HH model at $\beta = 20$ is given in Supplementary Fig. 9a. At such a low temperature, the system size has to be as large as $N = 36 \times 36$ to give an accurate finite-cluster description of the single-particle properties. A small $N = 4 \times 4$ cluster in Supplementary Fig. 9 would induce unphysical gaps in the $\langle n \rangle$ versus μ relation. Turning on a minimal magnetic field can provide a much better approximation despite the small effect from the magnetic field, as shown in Supplementary Fig. 9a. An alternative way is to conduct a DCA simulation. Since the dynamical mean field approximates the degree of freedom in the infinite bulk lattice outside the cluster, even an $N = 2 \times 2$ DCA cluster can represent the thermodynamic limit in describing the $\langle n \rangle$ versus μ relation, as shown in Supplementary Fig. 9b.

Now we turn on the interactions to $U = 8$. The recent exact diagonalization study[11] is a finite cluster calculation on a system size up to $N = 4 \times 3$ at zero field and zero temperature. DQMC simulations at a relatively high temperature $\beta = 8$ for cluster size $N = 3 \times 3$ and $N = 4 \times 3$ already shows considerable finite-size effects as shown in Supplementary Fig. 10(a) and (b) respectively, namely inducing a non-existing gap at $\langle n \rangle = 1$. The situation worsens at lower temperatures. In Supplementary Fig. 10c, the DCA simulation on an $N = 2 \times 2$ cluster benchmarks well with DQMC results on a $N = 6 \times 6$ cluster at high temperature $\beta = 4$. In addition, it shows no indication of a gap opening up to temperatures as low as $\beta = 20$.

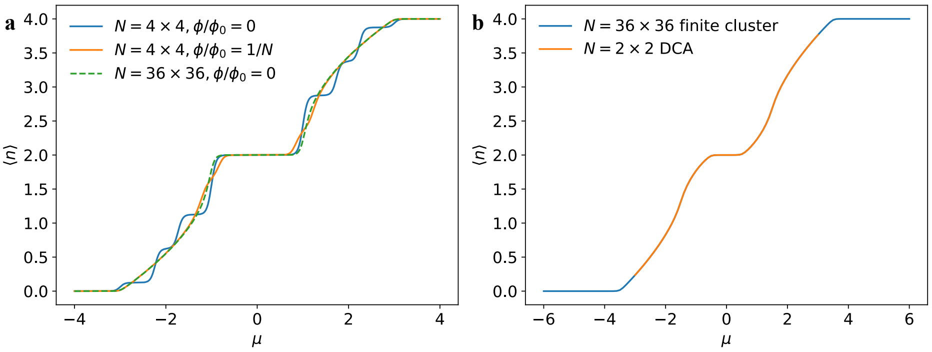

Supplementary Figure 9: $\langle n \rangle$ as a function of μ for the non-interacting BHZ-HH models at $\beta = 20$ from the calculations on different cluster sizes. **a** The comparison between $N = 4 \times 4$ (under zero and minimal finite field) and $N = 36 \times 36$ under zero field. **b** The comparison between $N = 36 \times 36$ finite cluster calculation and $N = 2 \times 2$ DCA simulations both under zero field. The parameter $M = 1$ for panel **a** and $M = 1.5$ for panel **b**.

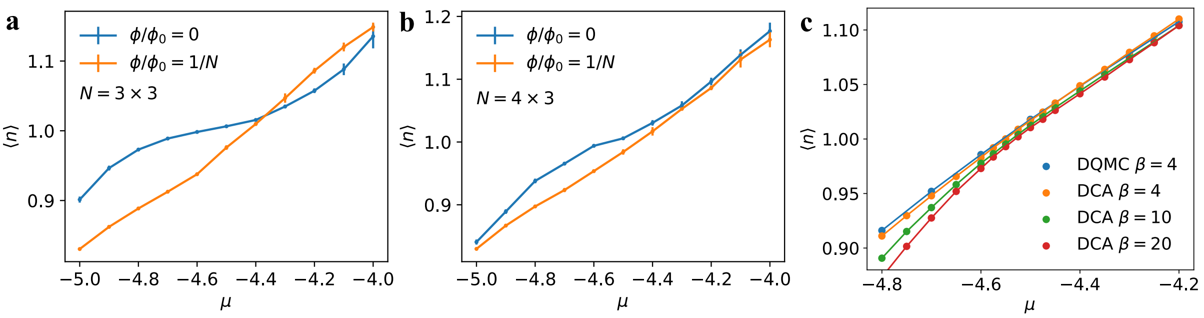

Supplementary Figure 10: $\langle n \rangle$ versus μ for the BHZ-HH models ($M = 1$) at $U = 8$ from DQMC and DCA simulations. Panels **a** and **b** are the DQMC results at minimal magnetic fluxes with $\beta = 8$ on a $N = 3 \times 3$ and $N = 4 \times 3$ cluster, respectively. Panel **c** includes DQMC results on an $N = 6 \times 6$ cluster with $\beta = 4$ and DCA results on an $N = 2 \times 2$ cluster with $\beta = 4, 10, 20$.

DQMC simulations for the BHZ-HH model at different U

The previous subsection showed that the gap barely opens in the DCA simulations for the BHZ-HH model ($M = 1$) at $U = 8$. Here from the compressibility plots in Supplementary Fig. 11, we find that the non-trivial topology already emerges at quarter-filling for an interaction strength as small as $U = 6$, thus justifying a semimetallic state with a high-temperature QSH feature. We also show the temperature-dependent inverse spin susceptibility at $U = 6$ and 8 in Supplementary Fig. 12. In either case, the spin susceptibility is unlikely to diverge at finite temperatures compared to the insulating case in Fig. 2a and Fig. 5d in the main text, supporting the semi-metallic state with no gap opening.

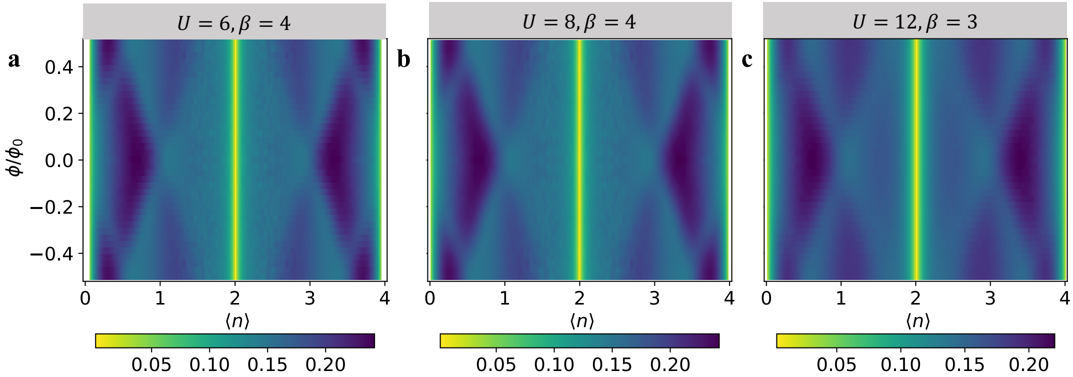

Supplementary Figure 11: The compressibility as a function of density and magnetic flux for the BHZ-HH model ($M = 1$) at **a** $U = 6, \beta = 4$, **b** $U = 8, \beta = 4$ and (**c**) $U = 12, \beta = 3$.

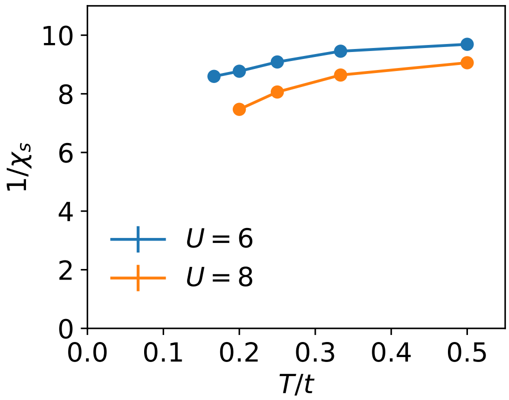

Supplementary Figure 12: The inverse spin susceptibility as a function of temperature for the BHZ-HH model ($M = 1$) at $U = 6$ and $U = 8$.

Finite-size analysis on the BHZ-HH model

To corroborate our finding of QSH effects driven by Mottness at quarter-filling, we conducted a finite-size analysis on the TRI compressibility (Supplementary Fig. 13(a)) and the compressibiility (Supplementary Fig. 13(a)) of the BHZ-HH model (Supplementary Fig. 13(b)) at low hole and electron density respectively for a range of filling that covers the feature from $3/4$- and $1/4$-fillings with $U = 12$. In Supplementary Fig. 13(c), we present the TRI compressibility at different magnetic fluxes $\phi/\phi_0 = 4/36, 8/36, 16/36$ and system sizes $N_{\text{site}} = L^2$ with $L = 6, 9, 12$. The collapse of the curves regardless of system size (panels (a) and (b)) suggests that the dip feature at $1/4$- and $3/4$-filling is fundamental and survives the thermodynamic limit. As a consequence, it makes sense to extract a spin Chern number. We find that for all the system sizes studied, $C_s = 1$ as depicted in Supplementary Fig. 13(c).

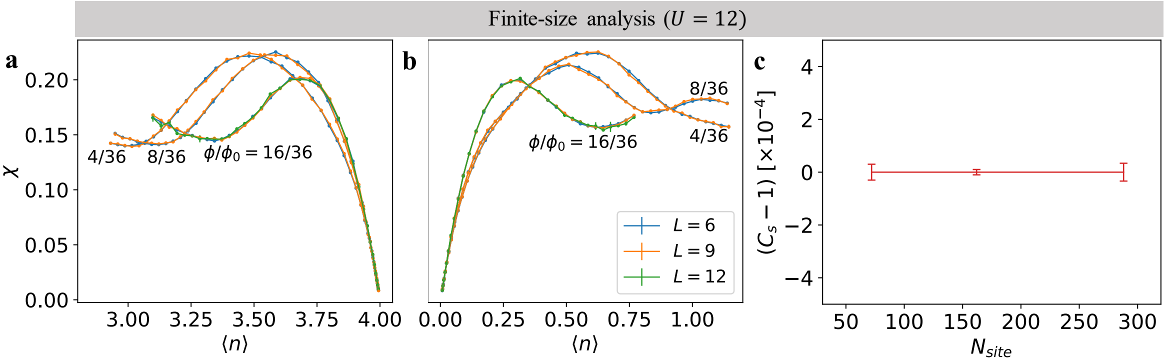

Supplementary Figure 13: Panels **a** and **b** show the TRI compressibility and compressibility respectively at low hole and electron density for the BHZ-HH model, at varying cluster sizes $N_{\text{site}} = L \times L$ under different magnetic fluxes as labeled. They share the same legend. Panel **c** presents the spin Chern number extracted for different cluster sizes. The temperature is $\beta = 3/t$.

Lower temperature results for BHZ-HH model

To explore lower-temperature physics, we are restricted to low density and a weaker interaction $U = 8$. In Supplementary Fig. 14, we show the TRI compressibility and compressibility at $\phi/\phi_0 = 16/36$ for the BHZ-HH model, though at low hole and electron density respectively, enough to capture the interested feature from quarter-fillings. In either case, the dips (representing the $C^{\text{TRI}} = 1$ valley for the TRI compressibility in Supplementary Fig. 14(a) and the zero-mode Landau levels for the compressibility in panel Supplementary Fig. 14(b)) don't decrease evidently despite the metallic peaks nearby rising up. This observation also supports a semimetallic state.

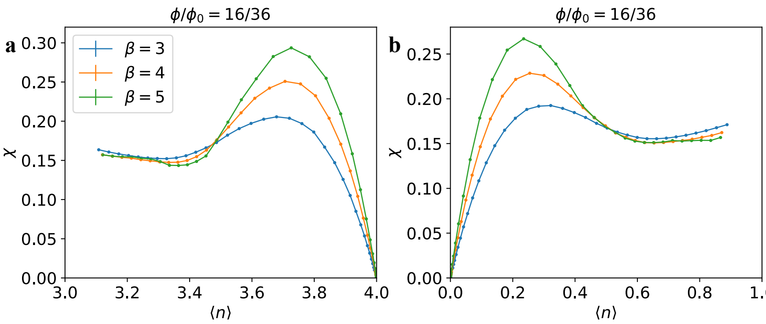

Supplementary Figure 14: The TRI compressibility (**a**) and compressibility (**b**) at low hole and electron density of the BHZ-HH model respectively, under a magnetic flux $\phi/\phi_0 = 16/36$ with different inverse temperatures $\beta = 3, 4, 5$. The interaction strength is $U = 8$.

Real-space spin correlation at half-filling under strong correlation

In the presence of strong correlation, at half-filling the non-interacting QSH order is destroyed and turns into a topologically trivial Mott insulator. We expect the Mott insulator to show anti-ferromagnetism (AF) robust to an external magnetic field in a bipartite lattice. However, Fig. 3(c) in the main text shows a peak in the spin correlation ($\mathbf{Q} = 0$) at half-filling, which is inconsistent with this expectation. To further explore this issue, we look into its zero-frequency spin correlation in real and momentum space at two temperatures $\beta = 3$ and $\beta = 5$, shown in Supplementary Fig. 15. Even at the relatively higher temperature of $\beta = 3$, the system already shows an AF pattern in the central region. As the temperature decreases, the AF region enlarges and further dominates the cluster. Thus, eventually at low enough temperature, AF order prevails in the Mott insulator, as expected. Note that here the smallest magnetic flux $\phi/\phi_0 = 1/36$ is used to reduce the finite-size effect at lower temperatures [4]. This does not affect our conclusion because the influence from the magnetic field is negligible compared to the AF order.

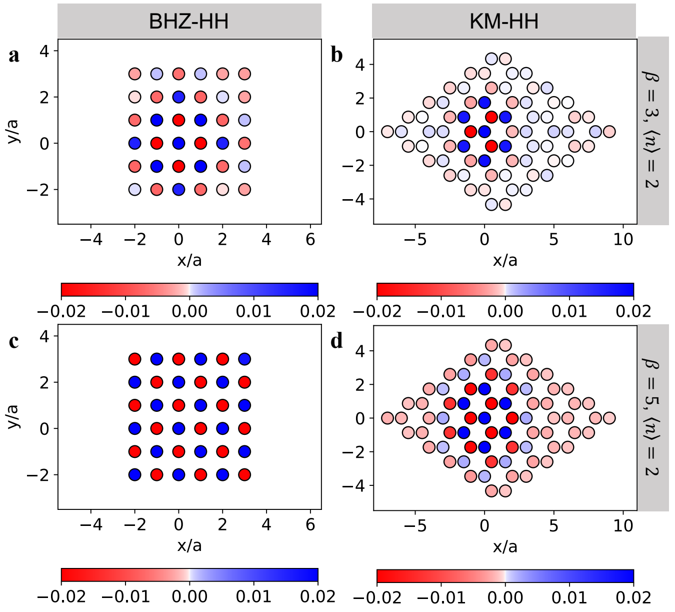

Supplementary Figure 15: The static spin correlation at half-filling ($\langle n \rangle = 2$) in real spaces respectively for BHZ-HH (**a**, **c**) at $M = 1$ and KM-HH (**b**, **d**) models at $\psi = 0.5, t' = 0.3$ both with $U = 12$ (in the model-specific energy scale) and zero magnetic field. The inverse temperatures are $\beta = 3$ for panels (**a**, **b**) and $\beta = 5$ for panels (**c**, **d**).

CALCULATION OF SPIN SUSCEPTIBILITY IN THE PRESENCE OF HATSUGAI-KOHMOTO INTERACTION

The BHZ-Hatsugai-Kohmoto (HK) model is

$$H = \sum_{\mathbf{k},\sigma} \left[(\varepsilon_{+,\mathbf{k},\sigma} - \mu)n_{+,\mathbf{k},\sigma} + (\varepsilon_{-,\mathbf{k},\sigma} - \mu)n_{-,\mathbf{k},\sigma}\right] + U\sum_{\mathbf{k}}(n_{+,\mathbf{k},\uparrow}n_{+,\mathbf{k},\downarrow} + n_{-,\mathbf{k},\uparrow}n_{-,\mathbf{k},\downarrow}). \tag{1}$$

We are mostly interested in zero temperature, strong correlations and low filling up to $1/4$. So then the singly and doubly occupied topological upper band is completely unoccupied. We can drop that for the current calculation. Adding a small Zeeman field, the low energy effective Hamiltonain becomes

$$H = \sum_{\mathbf{k},\sigma} \left[(\varepsilon_{-,\mathbf{k},\sigma} - \mu)n_{-,\mathbf{k},\sigma}\right] + U\sum_{\mathbf{k}} n_{-,\mathbf{k},\uparrow}n_{-,\mathbf{k},\downarrow} - h\sum_{\mathbf{k}}(n_{-,\mathbf{k},\uparrow} - n_{-,\mathbf{k},\downarrow}). \tag{2}$$

Note that $h\sum_{\mathbf{k}}(n_{-,\mathbf{k},\uparrow} - n_{-,\mathbf{k},\downarrow}) = h\sum_{\mathbf{i}}(n_{-,\mathbf{i},\uparrow} - n_{-,\mathbf{i},\downarrow})$. In Eq. (2), $\mathbf{k}$ is still a good quantum number. Then we can write down the partition function:

$$Z = \prod_{\mathbf{k}} Z_{\mathbf{k}},\;\; Z_{\mathbf{k}} = 1 + \exp[-\beta(\varepsilon_{-,\mathbf{k}} - \mu - h)] + \exp[-\beta(\varepsilon_{-,\mathbf{k}} - \mu + h)] + \exp[-\beta(2\varepsilon_{-,\mathbf{k}} - 2\mu + U)] \tag{3}$$

in which $\varepsilon_{-,\mathbf{k}} = \varepsilon_{-,\mathbf{k},\sigma}$ since the dispersion is independent of spin. Then the average occupation at each k and σ is

$$\langle n_{\mathbf{k},\sigma}\rangle = \frac{1}{Z_k}(\exp[-\beta(\varepsilon_{-,\mathbf{k}} - \mu - \sigma h)] + \exp[-\beta(2\varepsilon_{-,\mathbf{k}} - 2\mu + U)]) \tag{4}$$

The magnetic moment is

$$\langle m\rangle = \frac{1}{2}(\langle n_\uparrow\rangle - \langle n_\downarrow\rangle) = \frac{1}{2}\sum_{\mathbf{k}}(\langle n_{\mathbf{k},\uparrow}\rangle - \langle n_{\mathbf{k},\downarrow}\rangle) = \frac{1}{2}\sum_{\mathbf{k}}\frac{1}{Z_k}(\exp[-\beta(\varepsilon_{-,\mathbf{k}} - \mu - h)] - \exp[-\beta(\varepsilon_{-,\mathbf{k}} - \mu + h)]). \tag{5}$$

It is obvious that $\langle m\rangle \to 0$ as $h \to 0$, as expected. Next we calculate the magnetic susceptibility

$$\chi_s = \frac{d\langle m\rangle}{dh}\Big|_{h\to 0} = \sum_{\mathbf{k}} \frac{\beta\exp[-\beta(\varepsilon_{-,\mathbf{k}} - \mu)]}{Z_k(h=0)}. \tag{6}$$

From the main text, $\varepsilon_{-,\mathbf{k}} < 0$ for all $\mathbf{k}$ and $\mu = 0$ at $1/4$-filling. Then the magnetic susceptibility becomes

$$\chi_s(\langle n\rangle = 1) = \sum_{\mathbf{k}} \frac{\beta\exp[-\beta\varepsilon_{-,\mathbf{k}}]}{1 + 2\exp[-\beta\varepsilon_{-,\mathbf{k}}] + \exp[-\beta(2\varepsilon_{-,\mathbf{k}} + U)]} = \sum_{\mathbf{k}} \frac{\beta\exp[-\beta\varepsilon_{-,\mathbf{k}}]}{1 + 2\exp[-\beta\varepsilon_{-,\mathbf{k}}]}. \tag{7}$$

where at low temperature, the term $\exp[-\beta(2\varepsilon_{-,\mathbf{k}} + U)]$ is dropped for large U. At the zero temperature limit ($\beta \to \infty$),

$$\chi_s(\langle n\rangle = 1, \beta \to \infty) = \frac{A_B\beta}{2} \tag{8}$$

where A_B is the area of the Brillouin zone. This result can be generalized: for $\langle n\rangle \le 2$, $\chi_s(\beta \to \infty) \sim \beta$. To compare, the susceptibility for the tight-binding model is

$$\chi_s(\mu, U = 0) = \sum_{\mathbf{k}} \frac{\beta\exp[-\beta(\varepsilon_{-,\mathbf{k}} - \mu)]}{1 + 2\exp[-\beta(\varepsilon_{-,\mathbf{k}} - \mu)] + \exp[-2\beta(\varepsilon_{-,\mathbf{k}} - \mu)]}. \tag{9}$$

Setting $\mu = 0$ (half-filling $\langle n\rangle = 2$) and taking the zero temperature limit, it becomes

$$\chi_s(\langle n\rangle = 2, U = 0, \beta \to \infty) = \sum_{\mathbf{k}} \frac{\beta\exp[-\beta\varepsilon_{-,\mathbf{k}}]}{\exp[-2\beta\varepsilon_{-,\mathbf{k}}]}\Big|_{\beta\to\infty} \to 0. \tag{10}$$

The divergence of the spin susceptibility in Supplementary Eq. (8) indicates the instability towards a spin polarized ferromagnetic state, driven by strong correlation.

BILAYER KM MODEL

In Supplementary Fig. 16, comparing the schematic band structure of the KM model with that in the AB-stacked $MoTe_2/WSe_2$ heterobilayer[12] (blue and red colors are for Chen number $C = 1$ and -1 respectively), we find that the only difference is that they have opposite spin for the top Chern bands. This contrast leads to qualitatively different physics. Filling all four KM bands in Supplementary Fig. 16(a) gives rise to a trivial band insulator, while filling all four moiré bands in Supplementary Fig. 16(b) results in a double QSH insulator with a spin Chern number $C_s = 4$ since $C_\uparrow = 2$ and $C_\downarrow = -2$. The latter is inherently contradictory as non-trivial topology cannot result in the band insulator limit. Consequently, it is not possible to construct a four-band tight-binding model to describe this physics in AB-stacked heterobilayer. The principle for constructing a tight-binding model is that the system becomes a trivial band insulator if all bands are filled. Therefore, to describe the physics in the AB-stacked $MoTe_2/WSe_2$ heterobilayer, we use a bilayer KM model (with eight bands) whose Hamiltonian is

$$H = H_{\mathrm{KM}_1} + H_{\mathrm{KM}_2} + t_\perp \sum_{i,\sigma}(c^\dagger_{1i\sigma}c_{2i\sigma} + c^\dagger_{2i\sigma}c_{1i\sigma}) + V\sum_{i,\sigma}(c^\dagger_{1i\sigma}c_{1i\sigma} - c^\dagger_{2i\sigma}c_{2i\sigma}), \tag{11}$$

where i is the site label for each layer, σ represents the spin and the numbers 1 and 2 are layer indices, V is the voltage difference between layers. H_{KM_1} is the same as H_{KM_2} with only different layer indices. In the flat-band limit, the inter-layer hopping $t_\perp$ lifts the degeneracy of the bottom (or top) orbitals between two KM layers while the Chern number for each band with a specific spin is unchanged, as shown in Supplementary Fig. 17(a). Then the lower four bands are equivalent to the four moiré bands in the AB-stacked $MoTe_2/WSe_2$ heterobilayer (Supplementary Fig. 16) for which the valley labels can be assigned. Without interaction, this model shows a QSH effect at quarter-filling ($\langle n\rangle = 2$) and a double QSH effect at half-filling ($\langle n\rangle = 4$) in Supplementary Fig. 18(a-c) with an example flat-band parameter set $t'/t = 0.3$, $\psi = 2.54$, $t_\perp/t = 0.3$, $V/t = 0.4$ and $\beta = 12/t$.

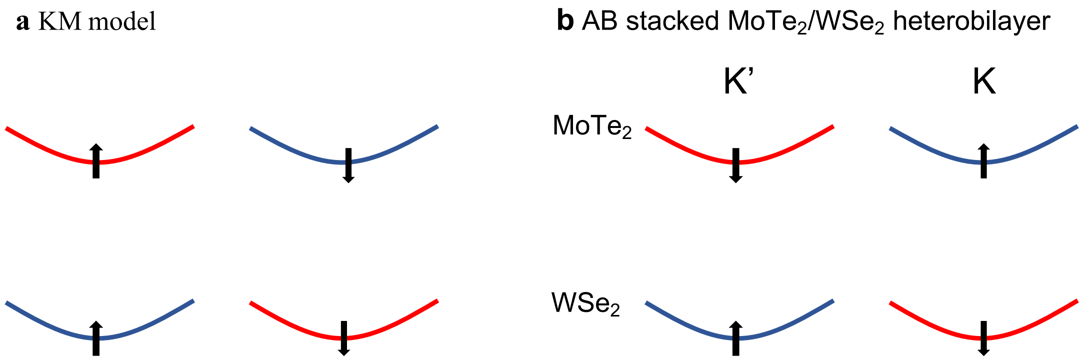

Supplementary Figure 16: The schematic band structures of **a** KM model and **b** AB-stacked $MoTe_2/WSe_2$ heterobilayer. Blue and red colors represent Chen number $C = 1$ and -1 respectively. K and K' are valley degrees of freedom.

We conduct the DQMC simulation for the corresponding bilayer flat-band KM-HH model at an intermediate Hubbard interaction $U = 1.5t, \beta = 12/t$ and present the compressibility, spin susceptibility and magnetization in Supplementary Fig. 18(d-f) respectively. The system exhibits a QAH effect with spin polarization at $1/8$-filling ($\langle n\rangle = 1$) and a QSH effect at quarter-filling ($\langle n\rangle = 1$), as expected given the similarity between single-layer and bilayer KM-HH models. Since the Hubbard interaction mixes the non-interacting bands and the resulting QAH state is spin polarized, this necessarily yields valley-coherence given the valley assignment in Supplementary Fig. 17(a).

The presence of an emergent topologically non-trivial state at $3/8$-filling ($\langle n\rangle = 3$) is special to the bilayer model. It has the feature of a QAH effect, namely the single Landau level in the compressibility (Supplementary Fig. 18(d)) accompanied by the peak in the spin susceptibility (Supplementary Fig. 18(e)). It is also likely to have the QSH feature. This obtains because the system can not be fully polarized at $\langle n\rangle = 3$. Some band must be doubly occupied, thereby explaining the white region in the magnetization in (Supplementary Fig. 18(f)). Also, the helical currents from the $\langle n\rangle = 2$ QSH state probably play a role in the $\langle n\rangle = 3$ edge state because their edge dispersion extends to the upper bands, which would not be filled until $\langle n\rangle > 4$. Therefore, for the state at $\langle n\rangle = 3$, we have either $C_\uparrow = 2$, $C_\downarrow = -1$ or $C_\uparrow = 1$, $C_\downarrow = -2$ depending on the polarization while the $\langle n\rangle = 1$ state has either $C_\uparrow = 1$, $C_\downarrow = 0$ or $C_\uparrow = 0$, $C_\downarrow = -1$.

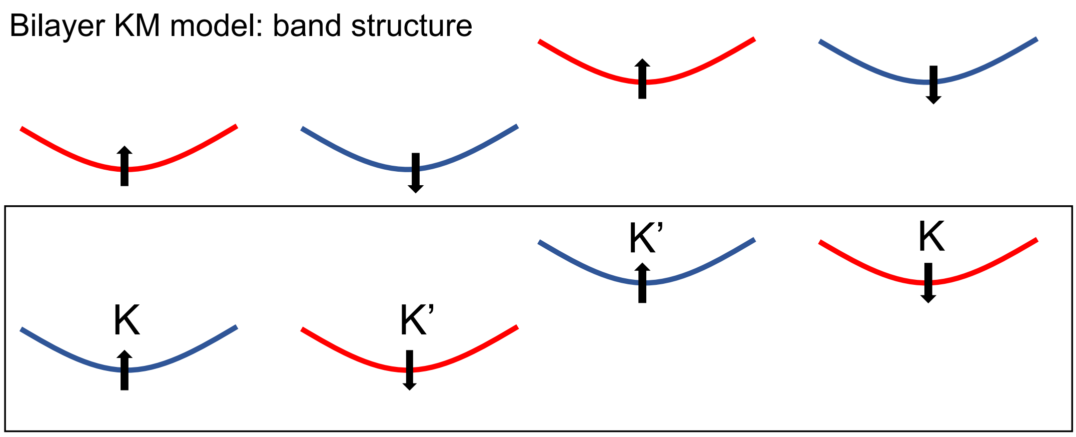

Supplementary Figure 17: The schematic band structure of the bilayer KM model. Blue and red colors represent Chen number $C = 1$ and -1 respectively.

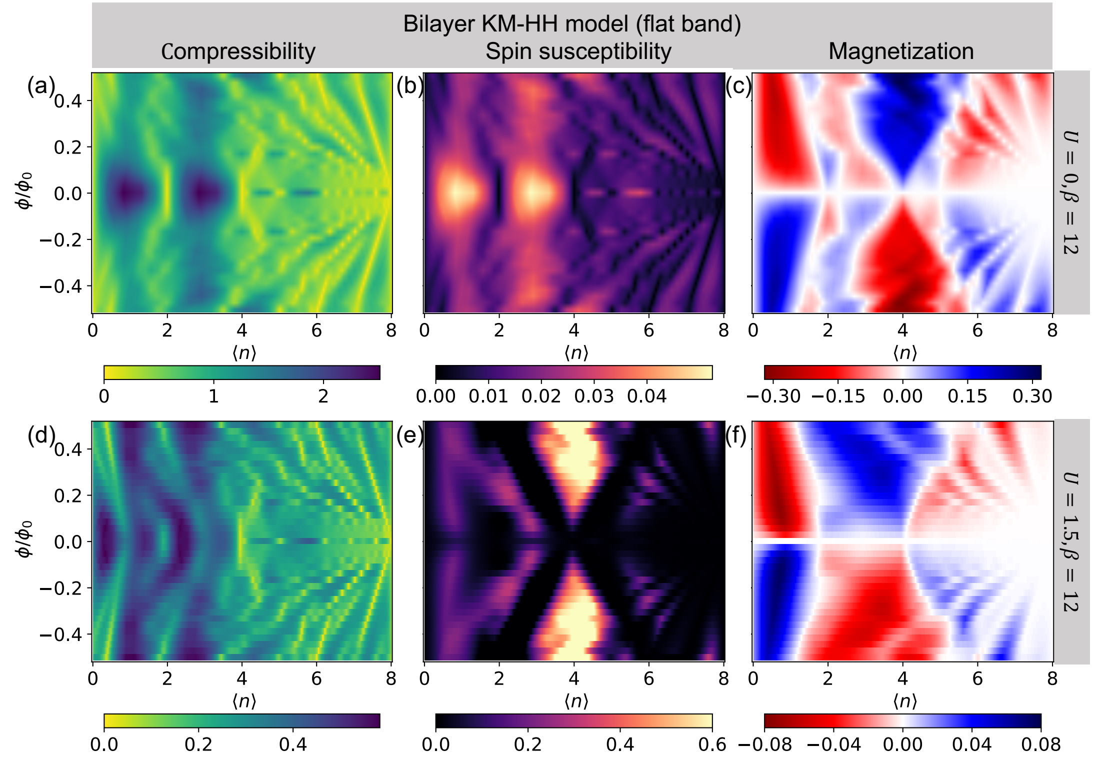

Supplementary Figure 18: DQMC results for the flat-band bilayer KM-HH models at $U = 0$ (first row) and $U/t = 1.5$ (second row). Each row shows the compressibility, spin susceptibility and magnetization, all as a function of density and magnetic flux. The parameters are $t = 1$, $t'/t = 0.3$, $\psi = 2.54$, $t_\perp/t = 0.3$, $V/t = 0.4$ and $\beta = 12/t$.

[1] U. R. Hähner, G. Alvarez, T. A. Maier, R. Solcà, P. Staar, M. S. Summers, and T. C. Schulthess, Computer Physics Communications **246**, 106709 (2020), ISSN 0010-4655, URL `https://www.sciencedirect.com/science/article/pii/S0010465519300086`.

[2] E. Gull, P. Werner, O. Parcollet, and M. Troyer, EPL (Europhysics Letters) **82**, 57003 (2008), URL `https://doi.org/10.1209/0295-5075/82/57003`.

[3] E. Gull, P. Staar, S. Fuchs, P. Nukala, M. Summers, T. Pruschke, T. Schulthess, and T. Maier, Physical Review B **83**, 75122 (2011), URL `https://link.aps.org/doi/10.1103/PhysRevB.83.075122`.

[4] P. Mai, E. W. Huang, J. Yu, B. E. Feldman, and P. W. Phillips, npj Quantum Materials **8**, 14 (2023).
[5] J. C. Budich, B. Trauzettel, and G. Sangiovanni, Phys. Rev. B **87**, 235104 (2013), URL https://link.aps.org/doi/10.1103/PhysRevB.87.235104.
[6] T. Yoshida, R. Peters, S. Fujimoto, and N. Kawakami, Phys. Rev. B **87**, 085134 (2013), URL https://link.aps.org/doi/10.1103/PhysRevB.87.085134.
[7] T. Yoshida, S. Fujimoto, and N. Kawakami, Phys. Rev. B **85**, 125113 (2012), URL https://link.aps.org/doi/10.1103/PhysRevB.85.125113.
[8] M. Hohenadler, Z. Y. Meng, T. C. Lang, S. Wessel, A. Muramatsu, and F. F. Assaad, Phys. Rev. B **85**, 115132 (2012), URL https://link.aps.org/doi/10.1103/PhysRevB.85.115132.
[9] M. Hohenadler, T. C. Lang, and F. F. Assaad, Phys. Rev. Lett. **106**, 100403 (2011), URL https://link.aps.org/doi/10.1103/PhysRevLett.106.100403.
[10] Z.-X. Li, Y.-F. Jiang, and H. Yao, Phys. Rev. Lett. **117**, 267002 (2016), URL https://link.aps.org/doi/10.1103/PhysRevLett.117.267002.
[11] Y.-C. Tzeng, P.-Y. Chang, and M.-F. Yang, Phys. Rev. B **107**, 155106 (2023), URL https://link.aps.org/doi/10.1103/PhysRevB.107.155106.
[12] Z. Tao, B. Shen, S. Jiang, T. Li, L. Li, L. Ma, W. Zhao, J. Hu, K. Pistunova, K. Watanabe, et al., *Valley-coherent quantum anomalous hall state in ab-stacked mote2/wse2 bilayers* (2022), URL https://arxiv.org/abs/2208.07452.
